# Fistula from left main coronary artery to pulmonary trunk

**DOI:** 10.1007/s12471-020-01405-1

**Published:** 2020-03-10

**Authors:** N. Papakonstantinou, N. Miaris, K. Argyrakis, S. Mitsiadis, A. Dimopoulos, G. Gavrielatos, N. Patsourakos, N. Kasinos, A. Theodosis-Georgilas, E. Pisimisis

**Affiliations:** Cardiology Department, “Tzaneio” General Hospital of Piraeus, 18536 Piraeus, Greece

## Answer

**Fig. 1:**
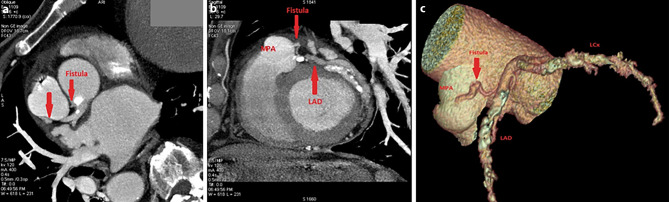
**a** and **b** Computed tomography coronary angiography views; **c** Three-dimensional reconstruction. A fistula arising from the left main coronary artery and draining into the main pulmonary artery is depicted. *LAD* left anterior descending artery, *LCx* left circumflex artery, *LMCA* left main coronary artery, *MPA* main pulmonary artery

The invasive coronary angiography showed a fistula originating from the left main coronary artery and no other haemodynamically significant coronary arterial lesions. Although the old age of our patient could discourage any further investigation (81-year-old patient with most probably a lifetime coronary fistula), computed tomography coronary angiography (CTCA) was performed and revealed this fistula draining into the main pulmonary artery (Fig. 1). Single-photon emission computed tomography with technetium-99m sestamibi showed permanent myocardial perfusion deficits with no stress ischaemic disturbances. Optimal medical treatment was adopted with good patient’s response.

Coronary-to-pulmonary artery fistulas are rare coronary connections (literature rates of <0.7%) most frequently originating from the left main coronary artery, the left anterior descending artery or the right coronary artery and draining into the main pulmonary artery [1]. Although they are often incidental findings (CTCA has increased diagnosis rates), patients may present with angina, dyspnoea, congestive heart failure, pulmonary hypertension, arrhythmias and sudden cardiac death. Therefore, their possible clinical effects need further investigation in order to adopt either interventional (surgery/transcatheter closure) or conservative treatment, avoiding any complications such as aneurysm creation, vessel dissection, pericardial effusion, coronary arterial steal phenomenon, thrombosis and myocardial infarction [1–3].
